# Numerical Modelling of Ballistic Impact Response at Low Velocity in Aramid Fabrics

**DOI:** 10.3390/ma12132087

**Published:** 2019-06-28

**Authors:** Norberto Feito, José Antonio Loya, Ana Muñoz-Sánchez, Raj Das

**Affiliations:** 1 Centre of Research in Mechanical Engineering—CIIM, Department of Mechanical and Materials Engineering, Universitat Politècnica de València, Camino de Vera, s/n, 46022 Valencia, Spain; 2 Department of Continuum Mechanics and Structural Analysis, University Carlos III of Madrid, Avda. Universidad 30, 28911 Leganés, Madrid, Spain; 3 Department of Mechanical Engineering, University Carlos III of Madrid, Avda. Universidad 30, 28911 Leganés, Madrid, Spain; 4 Sir Lawrence Wackett Research Centre, School of Engineering, RMIT University, 124 Latrobe Street, Melbourne, Victoria 3000, Australia

**Keywords:** aramid, impact, computational techniques, finite elements, mechanical analysis

## Abstract

In this study, the effect of the impact angle of a projectile during low-velocity impact on Kevlar fabrics has been investigated using a simplified numerical model. The implementation of mesoscale models is complex and usually involves long computation time, in contrast to the practical industry needs to obtain accurate results rapidly. In addition, when the simulation includes more than one layer of composite ply, the computational time increases even in the case of hybrid models. With the goal of providing useful and rapid prediction tools to the industry, a simplified model has been developed in this work. The model offers an advantage in the reduced computational time compared to a full 3D model (around a 90% faster). The proposed model has been validated against equivalent experimental and numerical results reported in the literature with acceptable deviations and accuracies for design requirements. The proposed numerical model allows the study of the influence of the geometry on the impact response of the composite. Finally, after a parametric study related to the number of layers and angle of impact, using a response surface methodology, a mechanistic model and a surface diagram have been presented in order to help with the calculation of the ballistic limit.

## 1. Introduction

With the aim of damage reduction in person subjected to ballistic impact, polymeric, carbon, and glass fibers are commonly used to develop protective systems. However, during last decades it has been found that a combination of high strength yarns in different directions generates flexible woven fabrics which are light and highly resistant at the same time [1]. Therefore, aramid fibers are one of the most used protection materials nowadays [2], with a growing trend in the industry.

Experimental studies have proved that the ballistic performance of aramid fabrics depends on many factors, such as the projectile geometry [3,4], the impact velocity [3,5,6,7], the friction between yarns [8,9,10], the woven structure of the fabric [6,11,12,13] and material properties [14]. However, most research has found that each property individually does not control the ballistic performance [15,16], rather the combined effect of all of these properties play a key role.

New studies to predict and to reduce the damage are constantly under study [17,18]. The use of surrogate models is common in optimal design problems to approximate the objective functions, as neuronal networks [19,20] or genetic algorithms [21]. However, a big dataset is needed in most cases to obtain good predicted values what can be expensive.

A useful tool for obtaining improved understanding of the behavior of aramid materials under impact loads is the use of numerical models. The ballistic limit, V_50_, the deformed shape of the woven fabric and the effect of the internal interlayer friction can be studied using the finite element method. However, the geometry of the fabric structure and the failure mechanisms of the yarns make the modelling of 3D fabrics complex [22,23,24,25,26,27,28,29,30]. To decrease the complexity of the finite element model, new simple models using shell elements [31,32,33,34,35] and truss elements [36] have been successfully implemented. 

Duan et al. [22], Rao et al. [23] and Grujicic et al. [29,30] developed 3D numerical models with solid elements representing yarns as homogenous continua. These studies analyzed mainly the friction coefficient between the yarns and the clamping conditions. Modelling results showed that the fabric boundary condition is a primary factor that influenced the friction effect, when only two edges are clamped, fabric reduces the residual velocity of the projectile and absorbs energy more effectively. Fabrics with high stiffness decelerate the projectile relatively rapidly meanwhile fabrics with high-strength yarns need more time to initiate the failure. The material which combines both properties is the most favorable of all the examined ones under the imposed boundary conditions considered in the study.

Despite the accuracy of 3D models, some researchers observed that they have two limitations. The first is that these models do not consider the statistical variability in the fabric geometry and material properties. The second one is the high computational cost due to the realistic representation of the fabric that requires a fine mesh with many elements. 

To address the first problem and improve the prediction of the V_50_ velocities, Nilakantan et al. [24,26,37] implemented a framework which incorporated the inherent statistical variability in the system. This framework can be used to calculate the probabilistic velocity response (PVR) curve for projectiles with different size and shape and for different clamped conditions. These studies analyzed the projectile geometry [26], the impact location sensitivity [27] and the fabric clamping conditions [28,38]. The findings showed that projectiles with a small impact area resulted in a higher residual velocity when the impact occurs in the gap between the yarns compared to when it occurs at the yarn crossover junction. The large cylindrical projectile, which had the flattest and the largest impact face, showed no sensitivity to impact location. The study also remarked that a circular shaped projectile shows lower dependency on the residual velocity with the projectile impact location than other configurations. This is mainly due to the fact that this configuration is not sensitive to in-plane fabric rotations, giving it a slight advantage over the other clamping configurations.

To address the second problem related to the computational efficiency, researchers presented different solutions. One option was the use of shell elements instead of solid elements [32,33,34,35]. Ha–Minh et al. [32,33] studied the effect of the number of elements used in the model (four and eight elements by yarn) and showed, in terms of velocity evolution, similar results in both cases. This is a significant conclusion because the computation time of the model with 8 elements is double than the model with 4 elements.

Chu et al. [35] studied mechanical properties of the yarns. It was concluded that the yarn density does not affect significantly the ballistic performance of the fabric. As opposed to this, it was observed that a high value of longitudinal Young’s modulus produces a faster deceleration of the projectile. 

The second solution to reduce the computational cost is to modify the original 3D model, generating a hybrid model, which is comprised of zones with different modelling resolutions and different finite element formulation, all coupled together with impedance matching interfaces [31,39,40,41,42]. Barauskas et al. [31] developed a model for a plain-woven single-ply. The fabric model presented three different zones: a zone close to the impact area including failure modelling, a second zone where the woven structure did not undergo failure, and a last zone (far from the impact area) implemented as orthotropic membrane. During this research, it was proved that it is not easy to validate this kind of approach due to the many different levels involved in the process.

The original model of Nilakantan et al. [26], was modified in later studies [39,40,41]. New models which combine solid and shell elements were tested under different distributions of both meso-level and macro-level in the fabric producing a low cost computational model. 

Finally, Ha-Minh et al. [42] carried out a study comparing three different hybrid models, that was a modification of their initial macroscopic-model. The numerical analysis showed that the difference among the models was negligible for results such as the evolution of projectile velocity and the global impact fabric behavior.

With the objective of studying the friction coefficient in aramid woven, Das et al. [36] implemented a model with truss elements. The study analyzed the influence of the friction coefficient and the clamping conditions. It was reported that there is a limit for the friction between yarns since the shape of the projectile is negligible and affects in a negative way to the energy dissipation and the failure mechanism.

In the present study, a simplified finite element model is developed using truss elements with the aim of establishing the advantage that 1D element models present over solid element models. A validation analysis proved that the simplified model is efficient in terms of computational cost and accuracy. A parametric study has been carried out using the numerical model, with specific focus on the effect of the impact angle, a parameter that is not usually analyzed in the literature. Results are used to generate a response surface diagram able to predict the ballistic limit for low impact velocities. This methodology will be a useful tool for rapid impact response analysis in the industry.

## 2. Theoretical Study

The study of the physical mechanisms by which impact pressure waves reach the chest and cause injury are continuously under study in the research community. It is a goal to determine how the impact pressure waves are transmitted to the thorax or the brain in order to implement effective preventive measures and reduce the exposure risks [43,44]. 

The energy absorption rate of aramid fabric during impact depends on many variables. Among all of them, it should be mentioned the fiber modulus and the material modulus, which is related to the ability to brake the projectile [22,45].

The breaking energy of the yarn is determined by its characteristics, such as tensile strength, elongation or modulus. These factors affect the transmitting velocity of the stress wave that is generated by the impact of the projectile. The wave must be dispersed rapidly, and the breaking energy must be as high as possible to increase the impact resistance and the ballistic limit (velocity at which the projectile passes through the material) [46].

The characteristics of the material, such as the interlacing geometry of the fibers, the thickness and the number of layers, also affect the distance at which the impact disturbance will have moved in a given time. They also influence the breaking energy of the material (ability to resist breakage due to an external force), which depends on the tensile strength and elongation of the material.

## 3. Numerical Model 

The numerical models implemented in this study were developed using the finite element explicit code ABAQUS. The following sections describe the methodology used to generate both models.

### 3.1. Projectile

**Figure 1:**
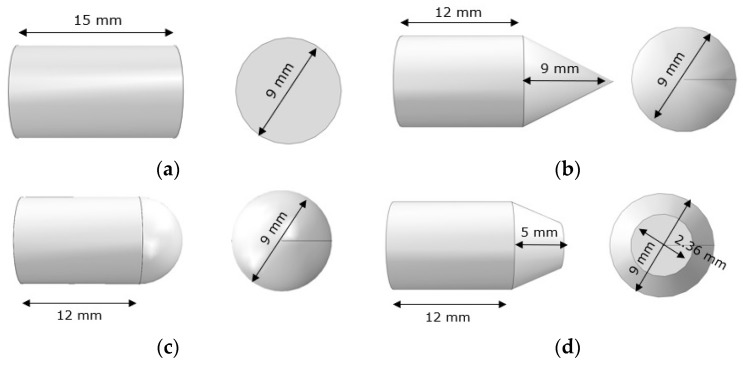
Dimensions of the projectiles: (**a**) blunt projectile, (**b**) conical point projectile, (**c**) hemispherical projectile and (**d**) truncated conical point projectile.

All projectiles were implemented as rigid solid objects to reduce the computational time [28]. Four geometric shapes (blunt, hemispherical, conical and truncated conical) was defined to keep the mass constant to 7.5 g for all cases (see Figure 1). The friction coefficient between projectile and material was established to be 0.22, taken from the literature [27]. As a rigid 3D object contained in a 3D space, a projectile has six degrees of freedom for motion. All rotation degrees have been restrained. The projectile can only traverse in the direction of the impact. The projectile velocity normal to the fabric is specified before each simulation and it ranged between 30–130 m/s based on experimental tests carried out in the same material by Yu et al. [47].

### 3.2. Specimen

**Figure 2:**
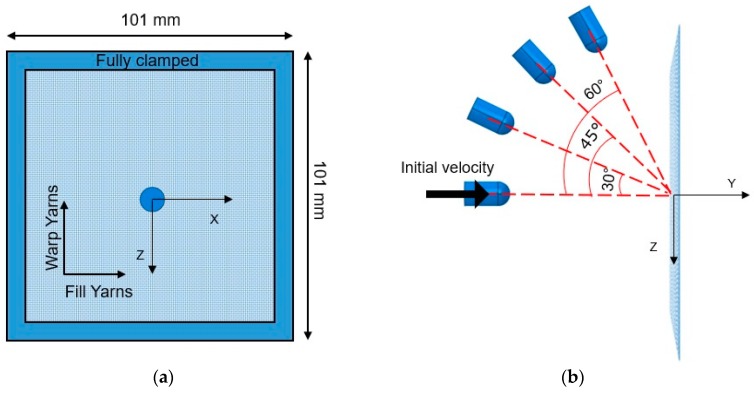
Fabric impact test setup with angular impact of the projectiles: (**a**) front view and (**b**) side view.

The specimen was impacted by the projectile in the center. It was fully clamped, resulting an effective area of 101 × 101 mm^2^ (Figure 2a). Each yarn had density of 1440 kg/m^3^ and was modelled as a linear-elastic material until failure to capture the behavior of the woven fabric subjected to ballistic impact (Figure 2b) [23,24]. When the ultimate strength was reached, the element was assumed to be damaged and removed from the computational domain. The mechanical properties of the aramid fabric were taken from the literature [36], where no apparent plastic deformation before fracture was reported. Table 1 shows the most important parameters of the yarns in both the directions. The static and dynamic friction coefficient values between yarns are 0.186 and 0.17, respectively. Both coefficients were obtained from semi-analytical model based on yarn pull-out tests [36].

**Figure 3:**
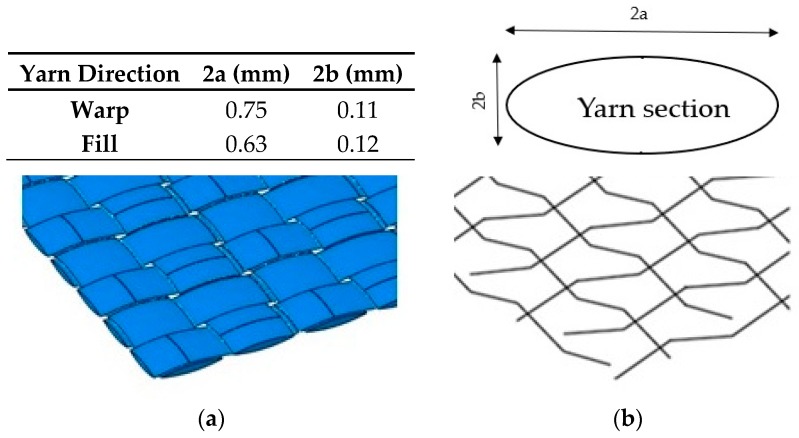
Yarn geometrical parameters. (**a**) Solid elements model and (**b**) Truss elements model.

The difference between both numerical models proposed, presented in Figure 3, is the representation of the yarns. In the complete full model case, the yarn dimensions were taken from [36] and were modelled with 3D solid elements (Figure 3a). The 3D elements were the standard volume elements of Abaqus. This element can be composed of a single homogeneous or heterogeneous material; they are more accurate if not distorted, particularly for quadrilaterals and hexahedra. In the present study the element used was C3D8R (eight-node linear brick) with reduced integration and hourglass control. It has three degrees of freedom by node [48].

On the other hand, the simplified model presents the yarns as 3D one-dimensional truss elements, which use linear interpolation for position and displacement, and have a constant stress. In this study, the element T3D2 (two-node linear displacement) was chosen. It had three degrees of freedom by node. Truss elements are long, slender structural members that can transmit only axial force and do not transmit moments [48]. The cross section was considered to be made up of trusses of an approximate cross section of 0.064 mm^2^ (Figure 3b). Dimensions of the yarns are also given in Figure 3.

## 4. Validation and Comparison between Models

**Figure 4:**
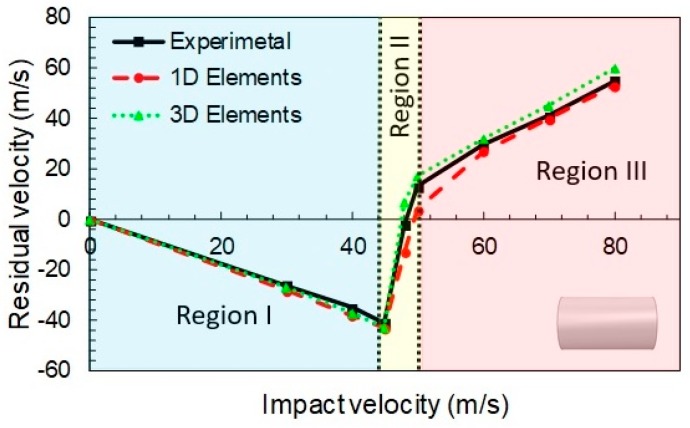
Calibrated ballistic curves for flat projectile. Experimental data taken from [36].

The study encompasses evaluation of computational time, projectile geometry effects and ballistic curve prediction (initial and residual velocities). For the flat nose projectile case, with and impact angle of 0°, the ballistic curves obtained with the 1D and 3D models proposed, and the corresponding experimental values published in [36], are presented in Figure 4. 

In the Figure 4, three different regions, typical from this kind of curves, are observed:

1. In the first one (*V_i_* < 44 m/s), the negative residual velocities indicate that the projectile rebounds due to the impact. This phase was dominated by fiber elongation. The yarns returned to the original configuration and released most of the elastic energy stored, imparting the projectile almost its original velocity towards the opposite direction. Thus, only a small amount of energy was dissipated.
2. In the second phase (44 m/s < *V_i_* < 52 m/s) an abrupt increment from a negative to a positive value of the residual velocity was observed. This corresponds to the rupture failure of the fabric. Some of the yarns surpassed their tensile yield stress and collapsed, dissipating energy in the process. The number of breaking (failed) yarns increased rapidly in a short range of velocities, allowing the projectile to pierce or penetrate the fabric.
3. If the initial velocity is further increased (*V_i_* > 52 m/s), the projectile always pierced the fabric, but the initial velocity was reduced by the dissipated energy. That is because the yarns first suffered an elongation, and then they failed by rupture because of the impact, absorbing some portion of the impact energy from the projectile.

The results obtained accurately reproduced the experimental trends above and below the ballistic limit. Here, negative residual velocities mean projectile rebound (impact velocity below the ballistic limit). In particular, the ballistic limit error was close to 4%, as can be seen in Table 2.

**Figure 5:**
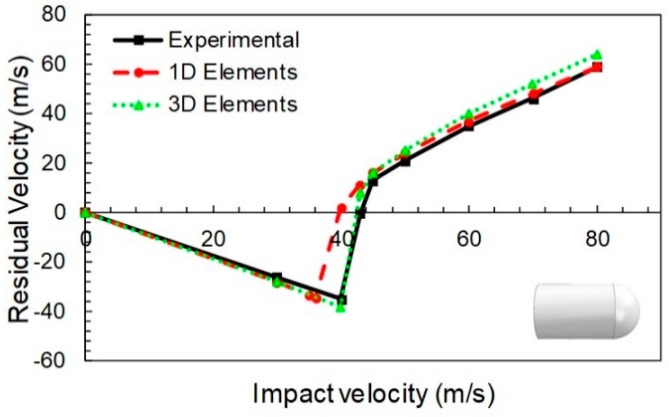
Validated ballistic curves for hemispherical point projectile. Experimental data taken from [36].

**Figure 6:**
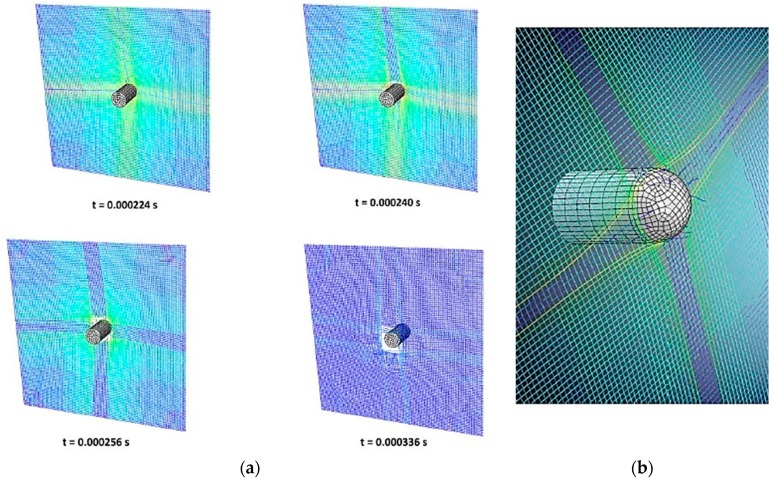
(**a**) Penetration of the hemispherical point projectile into a fully clamped layer at different time instants and (**b**) detail of the yarns failure.

Once the model was calibrated for the flat nose projectile, the hemispherical point projectile case was analyzed. Is this case, the general trends were also quite well predicted, see Figure 5. The three different regions described for the Figure 4 were also observed here. Although the error for the ballistic limit derived from the change of projectile geometry was close to −7% (Table 2), it can be considered acceptable according to the simplifications of the model and the level of engineering accuracy desired. Figure 6 shows the stressed fibers (typical cross section) at different instants of the simulation (Figure 6a). The fiber separation and fiber breakage during perforation is shown in Figure 6b.

In general, it can be observed that both 1D and 3D element models present reasonable accuracy when predicting the residual velocity of the projectile in terms of magnitude and trending. It is possible to observe the overestimation of residual velocity predicted with the solid model; in particular, it is slightly larger than that obtained with the simplified model, which involves that the solid model is conservative.

Despite an improved velocity estimation by the 3D model, the results obtained by the simplified model (which is around 99% faster, Table 3) lead to consideration it as an efficient tool in terms of computational requirement and accuracy level.

## 5. Results

After validating and comparing the results of both 1D and 3D element based models, a parametric study was carried out in order to analyze the influence of the projectile shape, the number of layers of the impacted panel and the impact angle. Even though both models are suitable to estimate the residual velocity, the 1D model was selected for the study because of its simplicity and low computational time. Results are discussed in the following sections.

### 5.1. Influence of the Projectile Geometry

In agreement with the literature related to low impact velocity in soft fabrics [47], the fabric area far from the impact zone, suffered a low deformation (a low displacement). Fixing the four sides of the fabric contributes to locate the stress and strain distributions around the impact area where the fabric generates a pyramid shape until failure.

**Figure 7:**
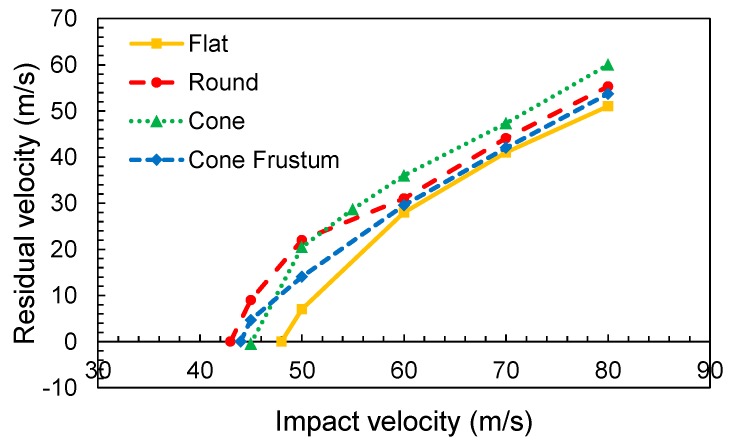
Ballistic curves for different projectile noses.

Figure 7 shows the evolution of the ballistic curves in terms of variation of the residual velocity with impact velocity for different projectile geometries. It can be observed that the flat nose projectile presents the highest ballistic limit, *V_BL_* = 49 m/s (velocity at which all impact energy is absorbed by the fabric and no penetration takes place). The round and truncated conical nose projectiles presented the same ballistic limit valued at 44 m/s, and the conical projectile at 46 m/s. The velocity value is reduced by 11% and 8% respectively, compared with the flat nose. This result implies that any sharper projectile presents a lower ballistic limit compared to a flat projectile [4]. This can be explained because the sharp projectile favors the separation of the transversal yarns generating a hole through which the projectile can penetrate the fabric. At the same time, the contact area was reduced to a smaller surface, causing greater stress. Under this situation, the fibers are more easily ruptured.

**Figure 8:**
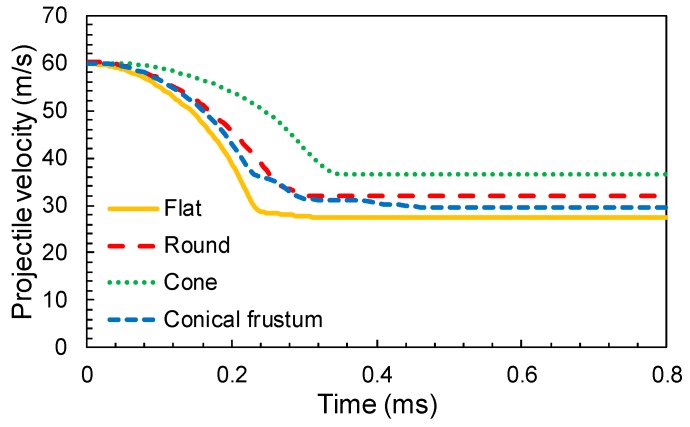
Typical velocity time history for different projectile geometries (*V_i_* = 60 m/s).

Figure 8 represents the projectile velocity evolution from the instant of impact moment. The initial velocity of the projectile fixed at 60 m/s is above the calculated ballistic limit. A few observations were derived from the relationship depicted. The cone geometry offers the lowest rate of deceleration while the rest of the geometries have relatively higher or similar rates. The projectile velocity histories of the round nose and truncated conical nose were quite similar, because the second geometry can be considered as a rough approximation to the round case. The energy dissipated by friction between the projectile and woven fabric increases with the frontal contact area, which was higher for the conical frustum projectile. Finally, the cone nose projectile has a higher residual velocity due to the sharp nose of the impact. This geometry favors the penetration of the fabric, as can be seen in [27].

**Figure 9:**
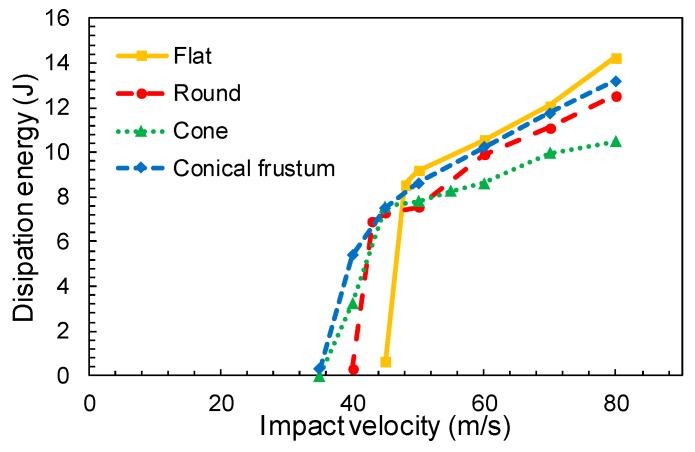
Variation of dissipated energy for different projectile geometries.

Based on the principle of energy conservation, the energy dissipated by the fabric (Figure 9) is equal to the loss of the projectile kinetic energy, given by Equation (1), where *m_p_*, represents the projectile mass, *V_i_* is the impact velocity and *V_r_* is the residual velocity.

(1) $$\Delta E=\frac{1}{2}m_{p}\left(V_{i}^{2}-V_{r}^{2}\right).$$

It can be observed in Figure 9 that the front face of the projectile plays an important role in the yarn breakage. While a sharp front face favors the separation of the yarns (cone nose projectile), the flat nose projectile penetration is entirely governed by yarn breakage which involves a higher load in the fibers and reduces the residual velocity [27,36].

### 5.2. Influence of the Impact Angle

The influence of the impact angle, defined as the angle between the projectile and the normal direction to the target (represented as α in Figure 2), was carried out with the round nose projectile. This projectile was chosen among the others because it is an intermediate geometry between flat and conic nose projectiles. It also has a close resemblance to common ammunition, like 9 mm “parabellun”. The velocity magnitude, *V*, was split into directions *Y* and *Z* as follows:

(2) $$\begin{array}{c} V_{Y}=V\cdot \cos\left(\alpha \right)\\ V_{Z}\;=V\cdot sin\;\left(\alpha \right) \end{array}$$

**Figure 10:**
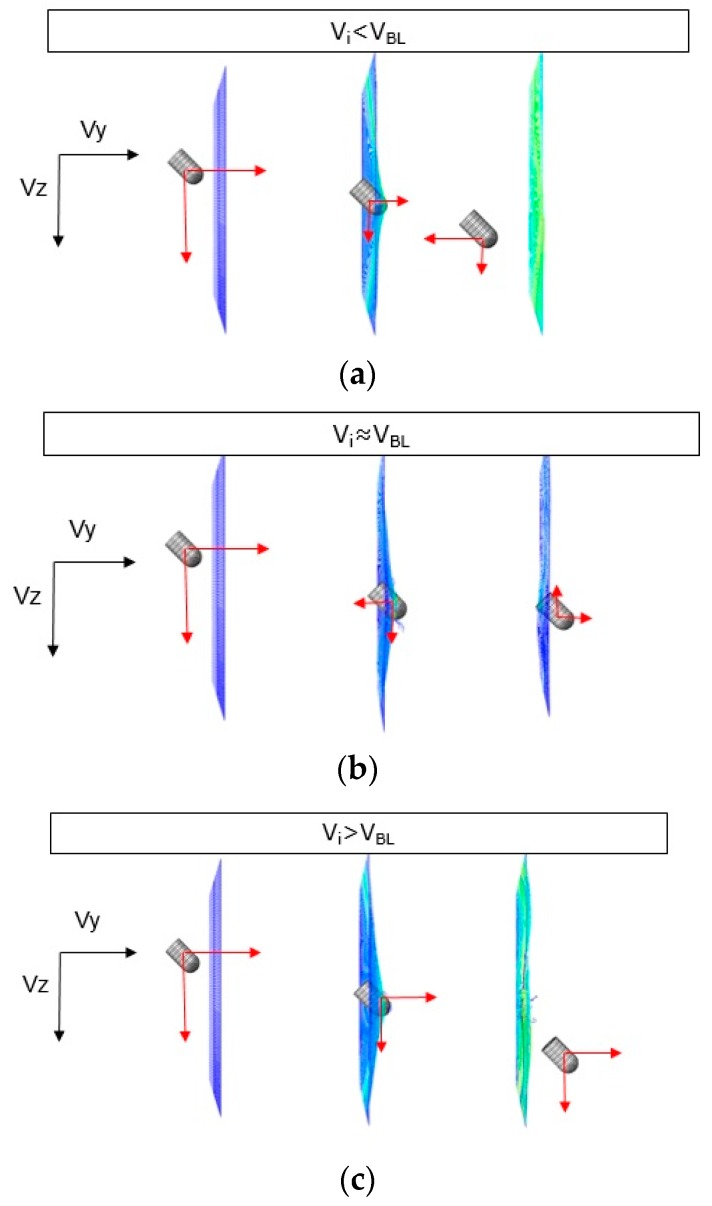
Schematic representation of *V_Y_* and *V_Z_* components as a function of the initial velocity *V_i_*. (**a**) Impact velocity below the ballistic limit, (**b**) impact velocity close to the ballistic limit, and (**c**) impact velocity above the ballistic limit.

**Figure 11:**
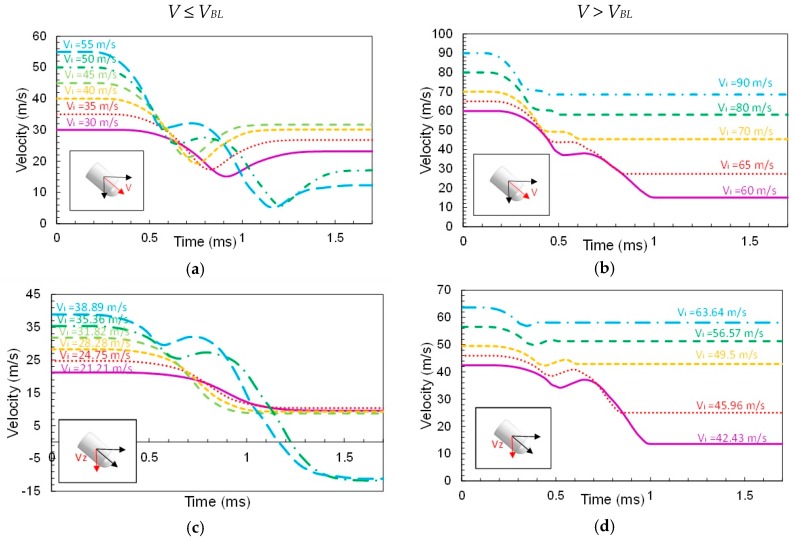

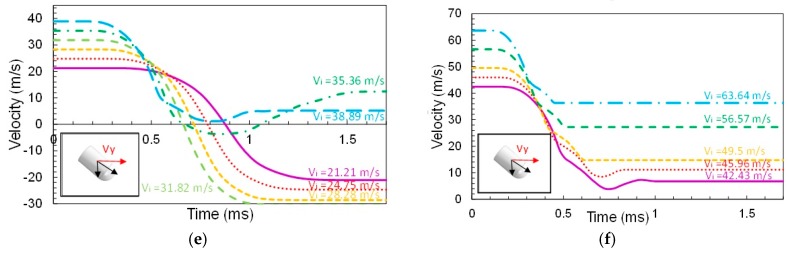
Velocity time histories for different initial velocities (impact angle = 45°): (**a**,**b**) magnitude of the velocity *V*, (**c**,**d**) velocity in the direction *Z* and (**e**,**f**) velocity in the direction *Y*.

For a particular oblique angle, *α* = 45°, three cases could be distinguished as function of the impact velocity (sketched in Figure 10 and detailed in Figure 11):

1. Below the ballistic limit, 30 m/s < *V* < 45 m/s: The projectile reduces its velocity components in both directions (*V_Y_* and *V_Z_*) during impact. The projectile changes the direction along *Y* axis because of the rebound (Figure 10a).
2. Close to the ballistic limit, 50 m/s < *V* < 55 m/s: The projectile suffers first a small rebound and then a small push out due to the wave generated in the fabric during the impact (Figure 10b).
3. Above the ballistic limit, 35 m/s < *V* < 60 m/s: The projectile continues its trajectory through the fabric with decreasing velocity in both the directions (Figure 10c).

Figure 11 illustrates the previous example. The left side plots correspond to impact velocities below the ballistic limit: the magnitude *V* (Figure 11a), *Y*-component *V_Y_* (Figure 11e) and Z-component *V_Z_* (Figure 11c). The right side plots correspond to impact velocities above the ballistic limit: the module *V* (Figure 11b), Y-component *V_Y_* (Figure 11f) and *Z*-component *V_Z_* (Figure 11d).

**Figure 12:**
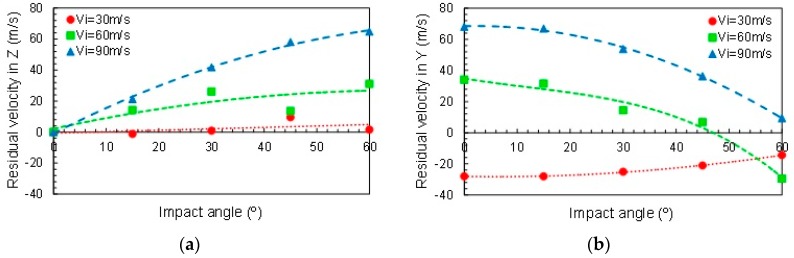
Residual velocity as a function of the impact angle (**a**) in *Z* direction (*V_Z_*) and (**b**) in *Y* direction (*V_Z_*).

The evolution of the residual velocity as a function of the impact angle is represented in Figure 12. Increasing the impact angle involves increasing the residual velocity in *Z* direction, due to the increase of *V_Z_* with the angle even in rebound cases (Figure 12a). Below the ballistic limit, rebound occurs for any angle (Figure 12b, *V_i_* = 30 m/s). Results show that the trend for *V_Y_* changes above the ballistic limit. It increases with the oblique angle, but at high angles the rebound may appear (*V_i_* = 60 m/s, α > 50°).

**Figure 13:**
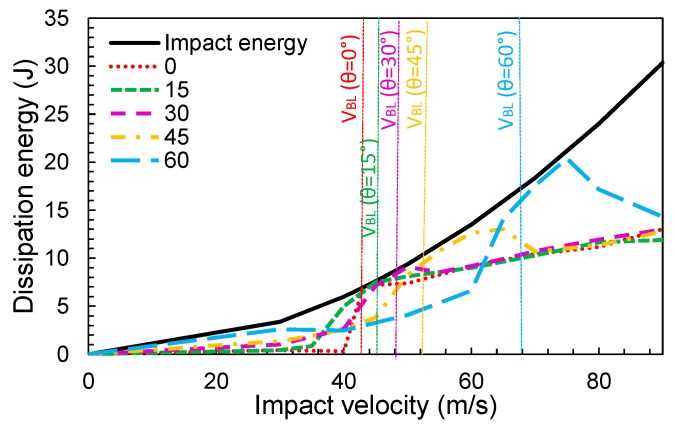
Variation of the dissipation energy with the impact velocity for different impact angles.

The evolution of the absorbed energy during impact for different angles is represented in Figure 13. Around the ballistic limit, the fabric absorbed almost all of the impact energy to decelerate the projectile. It is observed that the ballistic limit increases with the angle. Keeping the impact velocity constant (*V*), the kinetic energy component due to velocity in the *Y* direction decreased with the angle (Figure 11). In practice, the impact component in this direction is what produces the rupture of the yarns. Consequently, the projectile would need a higher initial velocity to break the yarns. It was found that increasing the angle to 66% leads to an increment of the ballistic limit of 58%.

**Figure 14:**
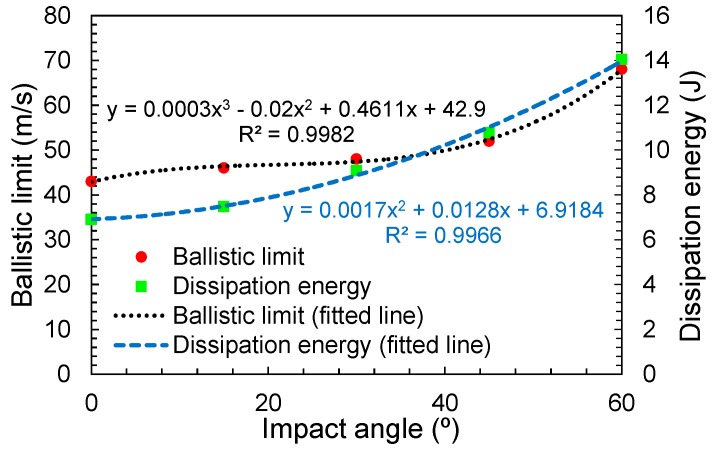
Variation of the ballistic limit and the dissipation energy with the impact angle.

For the hemispherical projectile, the relationship to estimate the dissipation energy and the ballistic limit as a function of the impact angle were calculated (Figure 14). For both cases, the *R^2^* value was greater than 0.9, which supports the validity of the equations.

### 5.3. Influence of the Number of Layers

The combination of the impact angle and the number of layers provides a multiple-choice problem. An initial study was carried out with the numerical model implemented and based on the round nose projectile. 

**Figure 15:**
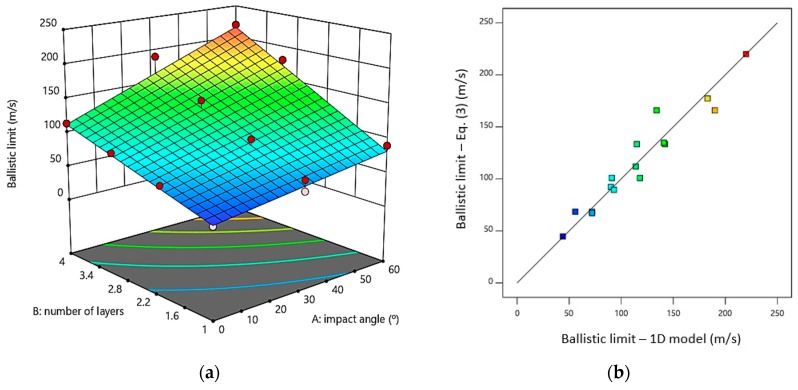
(**a**) Variation of the ballistic limit as a function of the number of layers and the impact angle; (**b**) correlation of the ballistic limit between the estimated values using the 1D model versus values estimated from Equation (3).

A fitted equation calculated with the results obtained from the numerical model is presented in Equation (3), where *n* represents the number of layers and *θ* is the impact angle. The *R^2^* value was 0.92, highlighting the validity of the fitted model. The mechanistic model shows a good correlation between the ballistic limit obtained with the numerical model and the ballistic limit calculated from Equation (3) as can be seen in Figure 15b.

(3) $$V_{BL}=22.250+0.458\cdot \theta +22.425\cdot n+0.335\cdot \theta \cdot n\;R^{2}=0.927$$

Figure 15a shows a response surface based on Equation (3). This Figure shows how the ballistic limit is enhanced by increasing the number of layers in the material. The ballistic limit value increases by 48%, 109% and 159% for two, three and four layers respectively. On the other hand, increasing the impact angle, raises the ballistic limit by nearly 27%, 63% and 95% for angles of 30°, 45° and 60° respectively. The evolution of the ballistic limit can be estimated rapidly using the fitted equation.

## 6. Conclusions

Based on the work presented in this paper, the following main conclusions can be drawn:

- A simplified model to study the impact in aramid fabrics at low velocities is been developed and parameterized. To validate the model, the results obtained were compared with the experimental tests reported in literature, obtaining a good agreement between the predicted values and the experimental results.
- The comparison of the 1D element based model with a 3D element based model demonstrated that the simplified models can reduce the computation time by 90%. This modelling methodology could be considered when designing personal protections with different woven structures and for various projectile geometries. The implementation of the numerical models in the industry, to help during the design process, requires simple and fast simulation tools.
- The computational analysis was also able to delineate the influence of different factors such as projectile geometry, number of layers and impact angle. Sharper projectiles lead to a higher residual velocity and a lower energy absorption, because the specific geometric feature of the projectile causes a higher deformation of the fibers allowing an improved slip through the fabric and facilitating rupture of the fibers. An increase in the impact angle and the number of layers lead to an increment of the ballistic limit.
- A mechanistic model developed for rapid estimation of the ballistic limit has been presented and validated with a very good confidence level. The expressions and surface diagrams obtained in this paper allowed to predict the critical velocity of impact once the number of layers and impact angle are known. This complementary analysis has elevated potential to be used in industry because of its simplicity. However, it is worth noting the necessity to carry out some previous work, both experimental and numerical, required to develop these types of mechanistic models with applicability in industrial environment.

## Figures and Tables

**Table 1:** Material properties of the Kevlar yarns [36].

Property	Warp Yarn	Fill Yarn
**Young Module (GPa)**	63.86	77.84
**Poisson’s ratio**	0.01	0.01
**Ultimate Strength (GPa)**	2.28	2.76
**Thickness (mm)**	0.11	0.12

**Table 2:** Ballistic limit results for the truss elements model.

Projectile Type	Experimental (m/s)	1D Model (m/s)	Error (%)
**Blunt projectile**	48	50	4.1
**Hemispherical projectile**	43	40	–6.9

**Table 3:** Comparison of computational time for the case of 60 m/s between 1D and 3D models.

Projectile Type	1D Model	3D Model	Computational Cost Reduction
**Blunt projectile**	480 s	33,213 s	98.55%
**Hemispherical projectile**	270 s	29,058 s	99.07%
